# Knowledge, attitude and seropositivity of hepatitis B virus among blood donors in the Bamenda Regional Hospital Blood Bank, Cameroon

**DOI:** 10.11604/pamj.2021.39.33.28911

**Published:** 2021-05-12

**Authors:** Moses Samje, Sylvain Sop, Claude Tagny Tayou, Dora Mbanya

**Affiliations:** 1 Department of Biomedical Sciences, University of Bamenda, Bamenda, Cameroon,; 2 Department of Clinical Sciences, University of Bamenda, Bamenda, Cameroon,; 3 Service d’Hématologie et de Transfusion, Centre Hospitalier et Universitaire (CHU), Yaoundé, Cameroon

**Keywords:** Hepatitis B virus, knowledge, attitude, blood donors

## Abstract

**Introduction:**

despite the existence of a preventive vaccine against hepatitis B viral (HBV) infection, approximately 250 million people are infected with the virus worldwide. This study aimed at evaluating the level of knowledge, attitude and seropositivity of the disease among apparently healthy, potential blood donors at the blood service of the Bamenda Regional Hospital Blood Bank.

**Methods:**

a cross-sectional study was carried out from March to May 2019 among 250 blood donors. Following screening for hepatitis B surface antigen (HBsAg) using the one-step HBsAg test strip, information on the level of knowledge and attitude towards the infection was obtained using a self-administered questionnaire. The correlation analysis was done to assess relationships between selected factors and knowledge of hepatitis B, p-value of 0.05 was considered as statistical significance.

**Results:**

the seropositivity of HBV was 6.4% (n = 16). Overall, 46.8% (n = 19) of the study participants had adequate knowledge while 76.3% (n = 31) had a positive attitude toward the disease. The highest seropositivity was observed in singles (7.1%; n = 13), primary school leavers (14.3%; n = 5), unskilled laborers (14.5%; n = 8) and replacement donors (9.33%; n = 7). The probability of being hepatitis B seropositive was higher in males, students (aOR: 8.8, 95% CI 0.7-96.1; p = 0.046) and those who had attained higher education (aOR: 3.2, 95% CI 0.8-12.7; p = 0.016). Independent factors responsible for higher odds of inadequate knowledge were being a male and attaining secondary education. On the contrary, students (aOR: 0.3, 95% CI 0.1-0.8; p = 0.012) and those with a history of blood donation (aOR: 0.5, 95% CI 0.2-0.9; p = 0.042) recorded lower odds of inadequate knowledge.

**Conclusion:**

the prevalence of hepatitis B among blood donors in this blood service is in the high intermediate category. Overall, the level of knowledge on this infection among these blood donors is average. These findings suggest that health education on HBV infection should be provided to the public as a major strategy to curb the infection.

## Introduction

Hepatitis B caused by hepatitis B virus (HBV) is a major global health problem and potentially a life-threatening liver infection. In 2015, an estimate of 257 million people were living with chronic hepatitis B infection and approximately 887,000 hepatitis-related deaths were recorded [1]. The highest prevalence of the infection is recorded in the Western Pacific and African regions [2]. Transmission is through contact with the blood or all other body fluids of an infected person. Although an effective vaccine for the virus has been available since 1982 [3], hepatitis B continues to be a serious public health problem.

Blood transfusion saves millions of lives yearly throughout the world and its demand is on the rise. Unfortunately, access to safe blood is a major problem mainly because of viral infections in the blood, notably, human immunodeficiency virus (HIV), hepatitis B virus and hepatitis C virus [4], in addition to blood shortage. Mbanya *et al*. [5] noted that only 80% of all donated blood is considered safe for distribution. Decades later, Virk and Hua reported a discard rate of 9% of blood in low-income countries due to the presence of infectious diseases in blood donation [6]. Among the transfusion transmissible infections (TTIs), HIV and HBV account for a higher proportion of discarded blood [5]. Even though the prevalence of HBV varies geographically, the rates remain high across most countries in Africa [4,7-10].

The level of knowledge and attitude toward hepatitis B infection fluctuates between different groups of respondents [11,12]. Among blood donors, general knowledge on the infection was 60% in a survey conducted at the Casablanca Regional Blood Transfusion Centre and 51.2% in North India with an attitude score of 47.93% [13,14]. Different studies conducted in some cities of Cameroon revealed differences in the level of knowledge on hepatitis B virus [15-17]. Although several studies have been conducted in Cameroon and Africa on the prevalence of HBV among blood donors, there is, nevertheless, sparse information on the level of knowledge and attitude of donors towards HBV. This study determined the seropositivity of HBsAg, evaluated general knowledge and attitude toward HBV infection among blood donors at the blood bank service of the Bamenda Regional Hospital, Cameroon to provide information in order to guide public policies in the fight against hepatitis B.

## Methods

**Study design and setting:** this was a cross-sectional study carried out from March to May 2019. The study recruited all potential blood donors who came to donate blood at the Bamenda Regional Hospital Blood Service. These study participants are representative of blood donors attending the blood bank service of this hospital. Before blood donation in this health facility, the medical history (including transfusion history) and vital signs of each potential donor are noted, and the donor undergoes both clinical examination and laboratory screening for TTIs. A donor is rejected from blood donation if there is an underlying medical condition or any risk associated with the donation. The admissible age group is 18 to 65 years with hemoglobin level ≥12.0 g/dl (females) and 13.0 g/dl (males). Female donors must not be pregnant, lactating, or menstruating. Pre-donation counselling is done to assess the suitability of everyone. The donor's response to the questionnaire is reviewed during a confidential interview with a trained staff member. Only donors who fulfilled these conditions and accepted to participate in the study by signing or thumb printing the informed consent form were recruited. All deferred donors were referred to the appropriate physician for further proper care management.

**Laboratory analysis:** a venous blood sample was collected from each eligible donor and analyzed for HBsAg using the one-step hepatitis B surface antigen test strip (Polymed Therapeutics Inc., Houston, USA). The test is based on the principle of a chromatographic immunoassay for the qualitative detection of the surface antigen of hepatitis B virus (HBsAg) in human whole blood, plasma and serum samples. Testing and interpretation of test results were carried out using the manufacturer's instructions. Prevalence was classified as low (<2%), low intermediate (2-4%), high intermediate (5-7%), or high (≥8%) [18].

**Instrument and scoring criteria of knowledge and attitude:** data on knowledge and attitude towards HBV infections were collected using a self-administered structured questionnaire. Where applicable, the questionnaire, which was originally in English was adapted and verbally administered in Pidgin English (broken English most widely spoken and understood language in the region) or French (one of the two official languages of the country). The questionnaire, which was divided into sections, generated information on the participant´s sociodemographic characteristics, donor's level of knowledge and attitude towards the infection. Questions on knowledge of HBV encompassed existence, mode of transmission, symptoms, treatment, prevention and control. In total, there were 33 questions on knowledge and each question was assigned a score of 1 for a total of 33 marks. A participant was deemed knowledgeable if the cumulative score was >50%. This flexible scoring system judged all with average knowledge as being knowledgeable. Scoring of attitudes was based on responses to seven questions encompassing the individual's perception of vulnerability to the infection, willingness to get tested and vaccinated and the mindset toward preventive strategies. Based on the responses, each correct response was attributed to a mark for a total of 7 marks. In like manner, attitude was considered good if a score of >50% was attained by the respondent.

**Statistical analysis:** data were analyzed using the statistical software IBM SPSS version 21.0 for windows. Continuous variables were checked for normality using the Kolmogorov - Smirnov (K-S) test. Differences in mean knowledge score were compared across different factors using independent t-test and one-way ANOVA. Multiple linear regression analysis was carried out to assess relationships between selected factors and knowledge of hepatitis B. We used logistic regression to determine factors associated with seropositivity and inadequate level of knowledge. All univariable with a p-value less than or equal to 0.05 were included in the final multiple logistic regression model. A two-tailed p-value of 0.05 was considered statistical significance.

**Ethics approval and consent to participate:** ethical clearance for the study was obtained from the Institutional Review Board of the University of Bamenda (2019/0033H/UBa/IRB) while administrative authorization was acquired from the Regional Delegation of Public Health for the North West Region (54/ATT/NWR/RDPH). Written informed consent was also obtained from all the study participants. The study was explained to all the participants and they were informed that participation was voluntary. Furthermore, they had the liberty to withdraw from the study at any time without any consequences on them. All the information obtained from the subjects were treated with the utmost confidentiality.

## Results

**Seropositivity of HBsAg among the study participants:** during the study period, a total of 494 participants visited the blood bank. Of these, 244 were rejected. Reasons for rejection were previous blood donation less than 3 or 4 months for males and females respectively (57), elevated blood pressure (45), women under menstruation (37), younger than 18 years (6) and refusal to provide consent (99). Finally, 250 participants were retained for the study, with males, replacement donors and those of the age group 18-25 years dominating (Table 1). The prevalence of HBsAg among blood donors was 6.4% (n=16). As detailed in Table 1, HBsAg was more frequent in males, singles, those who had attained only primary level of education, the unskilled and first-time donors.

**Table 1:** sociodemographic characteristics and seropositivity of HBsAg in 250 blood donors from the service of the Bamenda Regional Hospital Blood Service, March to May 2019

Variable	N (%)	HBsAg +ve	HBsAg -ve	Seropositivity (%)
Overall	250(100)	16	234	6.40
**Gender**				
Male	176(70.4)	14	162	7.95
Female	74(29.6)	2	72	2.70
**Age group (years)**				
18 - 25	130(52.0)	8	122	6.15
26 - 33	65(26.0)	4	61	6.15
34 - 57	55(22.0)	4	51	7.27
**Marital status**				
Single	183(73.2)	13	170	7.1
Married	65(26.0)	3	62	4.6
Divorced/widowed	2(0.8)	0	2	0.0
**Education**				
No formal education	2(0.8)	0	2	0.0
Primary	35(14.0)	5	30	14.3
Secondary	60(24.0)	5	55	8.3
Higher education	153(61.2)	6	147	3.9
**Occupation**				
Student	124(49.6)	7	117	5.6
Unskilled	55(22.0)	8	47	14.5
Employed	71(28.4)	1	70	1.4
**Residence**				
Rural	46(18.4)	2	44	4.3
Urban	204(81.6)	14	190	6.9
**Religion**				
Christian	224(89.6)	13	211	5.8
Muslim	19(7.6)	2	17	10.5
Pentecostal	7(2.8)	1	6	14.3
**History of blood donation**				
Yes	125(50.0)	5	120	4.0
No	125(50.0)	11	114	8.8
**Reason for donating blood**				
Replacement	150(60.0)	14	136	9.33
Voluntary	97(38.8)	2	95	2.06
Paid	3(1.2)	0	3	0.00

**Factors associated with seropositivity of HBsAg:** bivariate analysis of the factors associated with seropositivity of HBsAg are shown in Table 2. Males, secondary level of education and unskilled profession were all associated with HBV infection although the correlation was not significant. On the other hand, donors who had attained university level of education and students were significantly associated with infection [OR: 4.1, 95% CI 10.1 - 14.2, p=0.027 and OR: 11.9; 95% CI 1.4 - 98.4, p=0.021 respectively]. A greater proportion (11, 8.8%) of those who were donating blood for the first time were reactive to the HBsAg compared to participants with a previous blood donation history. The difference was, however, not statistically significant (Table 2).

**Table 2:** univariable and multivariable correlates of HBsAg seropositivity among blood donors

Parameters			Univariable analysis		Multivariable analysis	
		Reactive (%)	ORs (95% CI)	p-value	ORs (95% CI)	p-value
Gender	Female (74)	2 (2.7)	Ref			
	Male (176)	14 (8.0)	3.1 (0.7 - 14.0)	0.140		
Age group	18 - 25 (130)	8 (6.2)	Ref			
	26 - 33 (65)	4 (6.2)	0.836 (0.2 - 2.9)	0.778		
	34 - 57 (55)	4 (7.3)	0.836 (0.2 - 3.5)	0.807		
Marital status	Single (183)	13 (7.1)	Ref			
	Married (65)	3 (4.6)	Undefined			
	Widow/widower (2)	0 (0.0)	Undefined			
Education	No formal education (2)	0 (0.0)	Ref			
	Primary (35)	5 (14.3)	Undefined			
	Secondary (60)	5 (8.3)	2.2 (0.6 - 7.6)	0.201	6.6(0.8-8.8	0.193
	Higher (153)	6 (3.9)	4.1 (10.1 - 14.2)	0.027	3.2(0.8-12.7)	0.016
Occupation	Employed (71)	1 (1.4)	Ref			
	Unskilled (55)	8 (14.5)	4.2 (0.5 - 34.7)	0.185	4.6(0.5-44.0)	0.184
	Student (124)	7 (5.6)	11.9 (1.4 - 98.4)	0.021	8.8(0.7-96.1)	0.046
Residence	Rural (46)	2 (4.3)	Ref			
	Urban (204)	14 (6.9)	0.6 (0.1 - 2.8)	0.533		
Religion	Christian (224)	13 (5.8)	Ref			
	Muslim (19)	2 (10.5)	0.4 (0.04 - 3.3)	0.373		
	Pentecostal (7)	1 (14.3)	0.7 (0.05 - 9.2)	0.791		
Previous blood donation	No (125)	11 (8.8)	Ref			
	Yes (125)	5 (4.0)	0.4 (0.1 - 1.2)	0.130		

ORs= odd ratios; aORs= adjusted odd ratios; CI=confidence interval

**Level of knowledge on HBV infection among the study participants:** of the 250 participants, 117 (46.8%) had adequate knowledge of HBV infection, mode of transmission and preventive measures. Females were more knowledgeable (63.51%; n=47) than males (37.8%; n=70) and the difference was statistically significant (p<0.001). In like manner, significant differences were observed among age groups, level of education, occupation and reason for blood donation. When the per cent mean level of knowledge was assessed within each group, females, the age group of 18-25 years, those that had attained university education and students had a significantly higher level of knowledge compared to their counterparts within the group.

**Factors associated with inadequate level of knowledge:**Table 3 shows the factors related to inadequate knowledge. Males had a higher odd of having inadequate knowledge compared to females (OR: 2.1, 95% CI 1.1-4.1; p = 0.034; aOR: 1.8, 95% CI 1.0-3.4; p = 0.05). In the same vein, we observed that when compared to those who had attained higher education (college), those who had attained only primary and secondary education had higher odds of having inadequate knowledge. A significant difference was noticed with those who attained only secondary education level (aOR: 3.7, 95% CI 1.5-8.9; p = 0.004). On the contrary, students (compared to the employed) and those who had a history of blood donation (compared to those with no history of donation) recorded lower odds of inadequate knowledge, [aOR: 0.3, 95% CI 0.1-0.8; p = 0.012 (for students); aOR: 0.5, 95% CI 0.2-0.9; p = 0.042 (for those with a history of blood donation)].

**Table 3:** analysis of sociodemographic characteristics and blood donation history with level of knowledge among 250 blood donors from the service of the Bamenda Regional Hospital Blood Service, March to May 2019

Characteristics		Univariable analysis		Multivariable analysis	
		ORs (95% CI)	p-value	aORs (95% CI)	p-value
Gender	Male (176)	2.1(1.1-4.1)	0.034	1.8(1.0-3.4)	0.059
	Female (74)	Reference			
Age group (years)	18 - 25 (130)	0.8(0.3-2.1)	0.636		
	26 - 33 (65)	1.6(0.7-4.0)	0.291		
	34 - 57 (55)	Reference			
Marital status	Single (183)	3.9(0.2-74.7)	0.381		
	Married (65)	3.8(0.2-70.3)	0.368		
	Widow/widower (2)	Reference			
Level of education	No formal education (2)	Undefined			
	Primary (35)	1.7(0.5-5.8)	0.420	2.4(0.5-11.5)	0.271
	Secondary (60)	5.1(2.3-11.4)	0.000	3.7(1.5-8.9)	0.004
	Higher (153)	Reference			
Occupation	Student (124)	0.4(0.2-0.9)	0.020	0.3(0.1-0.8)	0.012
	Unskilled (55)	0.9(0.3-2.6)	0.778	0.7(0.2-2.6)	0.577
	Employed (71)	Reference			
Residence	Rural (46)	0.9(0.4-1.8)	0.701		
	Urban (204)	Reference			
Religion	Christians (224)	0.5(0.1-3.7)	0.535		
	Muslims (19)	0.4(0.04-3.6)	0.409		
	Pentecostal (7)	Reference			
Previous blood donation	Yes (125)	0.5(0.3-0.9)	0.043	0.5(0.2-0.9)	0.042
	No (125)	Reference			
Reason for donating blood	Replacement (150)	Reference			
	Voluntary (97)	Undefined			
	Paid (3)	Undefined			

ORs= odd ratios; aORs= adjusted odd ratios; CI=confidence interval

**Attitude of participants towards HBV infection:** this study examined the attitude of these potential blood donors toward HBV infection. Overall, the participants had a mean good attitude score of 76.3% (n=31). Slightly more than half (52.8%; n=132) of the participants think that anyone can acquire the infection. While 89.6% (n=224) are willing to do the HBV test, there is a higher percentage (90.8%; n=227) of those with a good attitude to taking the vaccine if tested negative. Considering the availability of a vaccine and assuming the participants have the means to pay for one only 75.6% (n=189) of the participants had a good attitude (Table 4).

**Table 4:** attitude of 250 potential blood donors from the service of the Bamenda Regional Hospital Blood Service, March to May 2019, towards hepatitis

Attitude	Responses	Frequency	Percentage
Do you think you can get hepatitis B?	Yes	132	52.8
	No	69	27.6
	I do not know	49	19.6
Should everyone be tested for hepatitis B?	Yes	215	86.0
	No	13	5.2
	I do not know	22	8.8
Will you like to take the test at any time?	Yes	224	89.6
	No	11	4.4
	I do not know	15	6.0
If your test is negative, will you like to take the vaccine if available?	Yes	227	90.8
	No	23	9.2
Assuming you have the means, will you be willing to pay for an HBV vaccine?	Yes	189	75.6
	No	61	24.4
Can you share household utensils with someone infected with hepatitis B?	Yes	119	47.6
	No	131	52.4
Will you ask for a new syringe or sharps before use?	Yes	229	91.6
	No	21	8.4

## Discussion

Transfusion transmissible infections such as HBV remains a significant threat to safe blood donation, especially in countries where the prevalence of these infections is high. For improved awareness and more efficient strategies to curb HBV infection to be employed, studies on the knowledge, attitude, and prevalence of the disease are necessary. This study evaluated the seropositivity, knowledge and attitude towards HBV infection amongst 250 blood donors at the Bamenda Regional Hospital Blood Service, which serves as a referral hospital for the entire North West Region of Cameroon.

In this study, the overall seropositivity of HBV infection was 6.4% (n=16). This high intermediate category observation on the overall seropositivity is lower than the reports of studies on blood donors in other African countries including Ghana (7.5% and 13.3%), Equatorial Guinea (10.01%), Cameroon (12.6%) [9,19-21]. Our findings are, nonetheless, higher than the recorded seroprevalence of 4.1% and 3.21 that was reported in respectively, Northwest Ethiopia and Madagascar [22,23]. Among first-time donors, our prevalence was higher than the 7.28% observed in Gabon [24] and lower than the 12.14%, 10.1%, 10.2% observed in respectively, Yaoundé-Cameroon [25], Edea-Cameroon [26] and far North-Cameroon [27]. Possible explanations for the relatively higher or lower seropositivity in this study include the differences in the epidemiology of HBV between different geographic regions, improvement in diagnostic technologies over the years with greater sensitivity and specificity, the economic status of the country and the increased sensitization about the disease. Another important explanation is the public health policies implemented in different countries regarding HBV prevention, vaccination, screening and treatment.

Like Tagny *et al*. [28] and Nwobegahay *et al*. [29] who recorded 71.7% and 70.4% males respectively for blood donation, our study population also had 70.4% males. This could be explained by the fact that most females are rejected during donor examination because of physiological changes such as menstruation, pregnancy and breastfeeding. The seropositivity was higher in males (7.95%) than in females (2.70%). The relatively higher seropositivity in males suggests other routes of exposure other than sex. This could be attributed to the use of unsterilized barbing razors to shave hair, outside socialization and multiple sex partners which is more common with males. Besides, there is the possibility that women have better access to screening, for example, prenatal screening.

HBV seropositivity was lower in those that had a history of previous blood donation compared to first-time donors. This is contrary to Jagannathan *et al*. who observed repeat donor status to be associated with HBsAg positivity [30]. Our observation in this study could be explained by the fact that those who donate blood repeatedly are counselled on TTIs thus creating awareness on the diseases. Therefore, such donors may tend to adopt a more careful lifestyle compared to first-time donors and this corroborates with the WHO recommendation for voluntary, regular, non-remunerated donors [31]. It is also certain that positive repeated donors do not return for blood donation or are excluded from blood donation.

This study also revealed that 46.8% (n=117) of our study population had adequate knowledge of hepatitis B infection. This result is slightly lower than those obtained by Boutayeb *et al*. and Bhasker *et al*. in Casablanca [13] and North India [14] who reported that 51.21% and 60% respectively of blood donors had good knowledge on hepatitis B. Again, it is lower than those seen in Nigeria [32] where 85% of the subjects had good knowledge on HBV. Our results are, however, similar to those of Tatsilong *et al*. [33] who reported 47% of health care workers as having good level of knowledge of HBV infection in Yaoundé, Cameroon. From the perspective of our study participants being all potential blood donors unlike the health care workers reported in [33], we may remark that general knowledge of HBV infection in this community is on the rise. This corroborates with increasing efforts on creating awareness about this infection in the community. We observed that participants in the age group 18-25 years were more knowledgeable as well as those who had attended higher education. This age category corresponds mainly to students. It further validates our observation on the positive relationship between a higher level of education and knowledge.

The higher the student goes into education, the more he/she is exposed to various channels of information on the disease and a high level of understanding as well. Although we did not stratify the various disciplines of education, we realized that this group included students in the medical field. Such participants are expected to be more knowledgeable considering that hepatitis B is part of their teaching curriculum [34]. When we considered the categories of donors, paid/voluntary donors were more knowledgeable than family/replacement donors. This could be explained by the fact that paid and voluntary donors probably donate blood regularly and are counselled on TTIs each time they come for donation. Family/replacement donors are seldom regular donors. Due to an emergency in most cases, such persons are sought to step in to provide blood for a relation. Some might be hearing about the infection for the first time during a pre-donation counselling session. This scenario might justify the low level of knowledge among this group of participants. As expected, those who had never participated in any health education program related to hepatitis B had a lower mean knowledge score compared to their counterpart that has taken part in one. This underscores the importance of more public education on the disease through various communication media and approaches to increase awareness.

When we evaluated attitude, the overall mean attitude score of 76.3% (n=191) was obtained. This result was higher compared to that obtained by Bhasker *et al*. [14] where only 47.1% of blood donors had a good attitude towards hepatitis B. Although the level of knowledge on HBV infection is slightly below average, the good attitude of these participants toward the disease is an indication of their willingness to know and prevent the disease. Though 90.8% (n=227) of the donors were willing to take the vaccine, only 75.6% (n=189) were ready to pay for the vaccine provided they have the means.

Some limitations admitted in this study include the recruitment of participants restricted to the venue of blood donation, which may not be representative of the total blood donors' proportion of the region. That notwithstanding, this is the first study to the best of our knowledge to assess blood donors' seropositivity, knowledge and attitude toward hepatitis B in the north west region of Cameroon. Since blood donation is a universal exercise that requires community participation; the results of our findings can be extrapolated to the situation in the community since these participants, coming from the community were recruited in an unbiased way.

## Conclusion

The prevalence of HBV infection among blood donors at the blood bank service of the Bamenda Regional Hospital is 6.4% (n=16). Males are at higher risk of being seropositive for HBsAg while those with a previous history of blood donation are at a lower risk. Overall, the level of donors' knowledge on HBV infection was slightly below average (46.8%, n=117). In total, 76.3% (n=189) of the donors have a good attitude towards the disease. We recommend that health education strategies about HBV infection be improved and adapted to provide control of transmission in the public.

### What is known about this topic

- Transfusion transmissible infections, such as hepatitis B virus renders blood unsafe for transmission, thereby, contributing to blood shortage;
- Effective utilization of the hepatitis B vaccine can increase the likelihood of safe blood for transfusion;
- Several factors affect the process of safe blood donation amongst which are the level of knowledge and attitude of the blood donors.

### What this study adds

- A novel study that has provided information on the seropositivity rate, level of knowledge and attitude toward HBV among potential blood donors in one of the major blood services in Cameroon;
- Determined the seropositivity rate of HBV in this blood service to be 6.4% (n=16) with 46.8% (n=117) of the potential blood donors having adequate knowledge;
- Adequate knowledge correlated with higher education, students and voluntary donors; overall, 76.3% (n=189) of the potential blood donors have a positive attitude toward HBV with 90.8% of those who have a negative HBV test willing to take the vaccine.
